# Annotations

**Published:** 1892-07-09

**Authors:** 


### Annotations.

**The Masculine Fringe.**

There is no doubt that the fringe across the forehead that is now so universal, is exceedingly becoming to the male. It imparts just that touch of delicate softness to the contour of the face which the revolutionised development of the sexes at the present time seems to call for. Our women are becoming stalwart in body and robust in mind; and coincidently our men are displaying a delicate diminution of muscle and a mild amiability of disposition which, when accompanied by a softly-whiskered face and gracefully fringed forehead, are quite irresistible. It has long been foreseen by women that a change of characteristics and places between them and men was the order of development of the near future. The men, too, have now discovered this; and the enthusiasm with which they have taken up their new rôle argues well for the peace of the domestic hearth, and the well-being of our future woman-governed estate. The new departure began, where it was inevitable that it should begin, among the cultured classes, and at Oxford and Cambridge more especially. But, so rapid is the spread of culture in these days, it has already extended to the middle and lower class. Let Allah be duly praised! The butcher's young man, calling the other day, had a fringe which was positively ecstatic—long, silky, shiny with beautifully-perfumed marrow, its ample flow concealing all his otherwise too obtrusive forehead, and its extremities kissing with touching affection the pimply skin stretched out over his nasal bones—he was truly a "thing of beauty," and to some muscular Phyllis he will doubtless be a "joy for ever." It was almost impossible to imagine anything more glorious even in the way of Greek gods. But a more striking object still awaited the writer that very morning in the person of the most juvenile of the porters at the railway station. To describe this youthful Hyperion, as sublimed by an incomparable fringe of the most copious magnitude, is impossible. Let it be understood that astonishment, not to say awe, was the prevailing sentiment among all the first-class passengers. They felt that their day was over. We cannot but be grateful—we are grateful, both for the rapid spread of such finished culture, and for the beautiful ease and grace with which even the largest-limbed of our masculine population are yielding to the growing and inevitable supremacy of the other sex.

**Mr. James Payn's Physicians.**

"In the thousand-and-one prescriptions I keep in my desk, there is not a word about enjoying oneself," writes Mr. James Payn in the *Illustrated London News*. Perhaps one hardly expects to find rules for pleasure laid down in a physician's prescriptions. But the suggestion contained in Mr. Payn's complaint against the doctors is not unworthy of a little consideration on their part. Mr. Payn seems to consider that the average doctor is a "Killjoy." "What helps to depress many of us," says this most genial of journalists, "is the wand of our medical advisers, which not only, as in poor Sancho Panza's case, removes all our favourite dishes from the board, but everything else which we take delight in, and especially tobacco." Stop, stop, Mr. Payn! You are surely running away with the harrows! The doctor, who is himself a "good fellow," holds that life without pleasure is hardly worth living, and such a doctor generally translates his private convictions into his spoken and written advice. One of the chief aims, indeed, of the modern physician is the dissipation of the melancholy which is often a principal feature of his patient's trouble; and the substitution for it of a more vivacious and wholesome state of things. We still believe, notwithstanding Mr. Payn's "honest doubts," that worry, ambition, and disappointment sour many tempers and impair the health of large numbers of contending Britons in these days of struggle. One of the first things the doctor advises in such cases is that life shall be taken more easily, and that a continual cheerfulness shall be substituted for a worrying anxiety. Not only is "a contented mind a continual feast," it is the very best of all prescriptions for efficient health and long life. We are quite horrified that Mr. James Payn should accuse physicians as a class of being enemies to pleasure. The modern rule, we are sure is—or, at any rate, ought to be—to prescribe both at the table and in the study, and, indeed, everywhere else, all things that make for pleasure—unless they are positively injurious. Taking the table as an example, one of the established principles of regimen with doctors is that a thing eaten with relish is more than half-digested. But we must stop short, or we shall find a treatise growing under our hands. Mr. Payn must look through his thousand-and-one prescriptions once more, and see if some of them are not capable of a more amiable interpretation than he has previously found in their crabbed hieroglyphics.

**A Paradise for Consumptives.**

A correspondent of the *Daily Chronicle* informs the public that he considers the Falkland Islands a "paradise for consumptives." He reports two cases of alleged cure, and intimates that he could give more. As consumptives, in many instances, feel themselves to be drowning, they are usually very ready to catch at the proverbial "straw." Before giving themselves the inconveniences of a voyage of several thousand miles to the Falkland Islands, they will do well to devote attention to two or three circumstances which make for an opposite conclusion to that arrived at by the correspondent of the *Daily Chronicle*. In the first place, it is obvious that two, or two dozen, cases of cure, even if they were scientifically verified, are insufficient to found a generalisation upon. But a second, and far more important consideration is that the climate of the Falkland Islands seems to be the exact opposite of that which modern medical science and experience have decided to be most suitable for consumptives. A clear, bracing, and dry atmosphere at a mountainous altitude is universally believed by modern consumption specialists to offer the most favourable climatic conditions for those who are the victims of tubercular phthisis. But the climate of the Falkland Islands, although it is said to be healthy and to resemble that of the Orkneys, is neither dry, nor particularly clear, nor specially bracing; and the country is certainly not mountainous. Its most marked characteristics are said to be the severity of its gales and the abundance of its moisture. The average number of rainy days is 240 in the year, more than four a week. Compared with this, our damp England is almost a dry Sahara. The average annual temperature in 1887 was 34°. There are no trees on the islands. The principal vegetation consists of tussock grass and balsam bog. The climate, in fact, probably resembles that of the moister and more melancholy parts of Ireland. We should certainly advise consumptives to choose the hilly parts of England and Southern Europe rather than the Falkland Islands, unless they can obtain more convincing evidence than that which is furnished by the *Daily Chronicle's* correspondent.

